# *Punica granatum L*.-derived omega-5 nanoemulsion improves hepatic steatosis in mice fed a high fat diet by increasing fatty acid utilization in hepatocytes

**DOI:** 10.1038/s41598-020-71878-y

**Published:** 2020-09-17

**Authors:** K. Zamora-López, L. G. Noriega, A. Estanes-Hernández, I. Escalona-Nández, S. Tobón-Cornejo, A. R. Tovar, V. Barbero-Becerra, C. Pérez-Monter

**Affiliations:** 1 Departamento de Gastroenterología, Instituto Nacional de Ciencias Médicas Y Nutrición Salvador Zubirán, Vasco de Quiroga 15, Sección XVI, Tlalpan, 14080 CDMX México; 2 Departamento de Fisiología de La Nutrición, Instituto Nacional de Ciencias Médicas Y Nutrición Salvador Zubirán, Vasco de Quiroga 15, Sección XVI, Tlalpan, 14080 CDMX México; 3 Unidad de Medicina Traslacional, Fundación Clínica Médica Sur, Toriello Guerra, Puente de Piedra 150, Tlalpan, 14050 CDMX México

**Keywords:** Hepatology, Liver diseases, Non-alcoholic fatty liver disease, Gastroenterology

## Abstract

Pomegranate seed oil (PSO) is mainly composed of punicic acid (PA), a polyunsaturated fatty acid also known as omega-5 (ω-5), a potent antioxidant associated with a variety of metabolic and cellular beneficial effects. However, the potential benefits of a nanoemulsified version of ω-5 (PSOn) have not been evaluated in a pathological liver condition. Here, we examined whether PSOn had beneficial effects on C57BL/6N mice fed a high-fat diet (HFD), specifically on hepatic steatosis. We observed that PSOn supplementation decreased body weight and body fat mass in control mice, whereas glucose intolerance, insulin resistance, energy expenditure, and hepatic steatosis were improved in both control mice and in mice fed a HFD. Interestingly, PSOn increased fatty acid oxidation in primary hepatocytes and antioxidant gene expression. Altogether, our data indicate that PSOn effectively reduces some of the HFD-derived metabolic syndrome indicators by means of an increase in fatty acid oxidation within hepatocytes.

## Introduction

Pomegranate fruit (*Punica granatum L*.) is originally from the Middle East and is grown in countries such as Argentina, Israel, and the USA, among others^1^. Pomegranates can be transformed into edible products such as juice, nectars, jellies, and seed oil, all of which might have therapeutic effects, such as anti-proliferative properties against skin, prostate or breast cancer cells^2–5^, and the improvement in insulin resistance^6^. Current investigations indicate that pomegranate’s therapeutic properties depend on several plant components, including anthocyanin, ellagic acid, tannins, ursolic acid, ellagitannins, sterols, and punicic acid, among many other by-products^7,8^, with potential antioxidant, anti-glycaemic, anti-inflammatory, and even antimicrobial properties^1,9,10^. Pomegranate seed oil (PSO) obtained by cold pressing seems to retain most of the plant’s beneficial properties. In an in vitro study, the activity of the rate-limiting enzymes cyclooxygenase and lipoxygenase, which produce the inflammatory mediators prostaglandins and leukotriene, respectively, were significantly inhibited upon exposure to PSO in comparison with fermented pomegranate juice^11^. Other reports indicate that PSO consists of a mixture of conjugated fatty acids (CFAs), and depending on the cultivar, punicic acid (PA) constitutes approximately 70% to 80% of that mixture^12^. PA, also known as trichosanic acid, is an omega (ω)-5 polyunsaturated fatty acid with the formula 18:3 cis-9, trans-11, cis-13. It is an isomer of conjugated α-linolenic acid (CLnA) with structural similarities to conjugated linoleic acid (CLnA) and α-linolenic acid (LnA)^13^. Numerous studies have indicated that CLnAs have positive effects on cardiovascular and bone health, as well as body composition^14^. As PA is a CLnA, its potential health benefits have also been tested both in animal models and in humans. In the first case, PSO is able to significantly reduce obesity, bone loss, or insulin resistance in mice fed a high-fat diet^6,15,16^, while in human clinical trials, the antioxidant and anti-inflammatory effects of PA have been associated with a decrease in prostate-specific antigen (PSA) expression or lipid peroxidation, as well as a downregulation of high-density lipoprotein (HDL) and low-density lipoprotein (LDL) aggregation, which might prevent cardiovascular events^17–19^. These studies have proposed that the antioxidant and anti-inflammatory properties of PA are responsible for the observed effects.

As different components of the pomegranate fruit have been tested for their beneficial health effects, new physicochemical modifications have been introduced in order to fulfil these properties and to maintain the stability and bioavailability of their biological active components as well. Colloidal silver nanoparticles of pomegranate peel extract, for example, had strong antioxidant, antibacterial, and anti-proliferative properties in vitro^20^, while a nanoemulsified suspension of the seed oil (PSOn) was recently tested in a murine model of Creutzfeldt-Jakob disease (CJD) in order to assess its bioavailability, its activity against prion protein aggregation, and its effects on neurodegeneration-associated pathological features. The major findings of the study were that, indeed, mice with CJD had significant delays in the onset of the disease, along with the reduction in the generation of reactive oxygen species (ROS) and a meaningful protection against neuronal death when treated with the PSOn emulsion compared to the non-emulsified PSO^21^.

However, there is no evidence whether PSOn could have beneficial effects on the metabolic disturbances induced by a high-fat diet. Therefore, we aimed to test whether PSOn is able to prevent or reverse metabolic markers of metabolic alterations in a high-fat diet-induced obesity murine model. Our study provides novel information concerning the use of PSOn as an alternative treatment to tackle metabolic syndrome and fatty liver disease.

## Results

### PSOn oral gavage administration has no detrimental effects on mice

As a first approach, we decided to perform toxicological experiments on mice to test whether PSOn has any harmful health risk on global homeostasis. In previous reports, other researchers have administered PSO or other organic oils by direct addition to chow diets; however, this strategy has some disadvantages due to the uncertainty of the total and most reliable amount of food consumption per mice. To overcome this problem, we decided to take advantage of the water solubility of PSOn and feed the mice with an oral gavage strategy. First, we tested the administration of three different single doses (1, 2 or 4 mg/g) of PSOn in 8-week-old C57BL/6N mice. Fifteen days later (acute protocol, see Supplementary Materials), peripheral blood and internal organs (liver, kidneys, among others) were obtained to perform histological and biochemical analyses. Mice did not show any signs of abnormal behaviour after oral administration or at the time of death. We did not find any gross disturbance in body weight gain or organ/body weight ratio (Supplementary Fig. 1A,B). In addition, liver and kidney histology did not reveal tissue damage at the cellular level (Supplementary Fig. 1C) or in the biochemical parameters (alanine-amino-transferase (ALT), aspartate-amino-transferase (AST), triglycerides, cholesterol, albumin, and glucose) (Supplementary Fig. 1D). In an independent experiment, we also evaluated the long-term effect of PSOn in an every-other-day administration basis (chronic protocol, Supplementary Materials). Behaviour, food consumption, and body weight were monitored and, as before, blood and tissues were collected. The same parameters were evaluated; however, we did not observe any histological or biochemical differences (Supplementary Fig. 2A–D). Thus, we concluded that PSOn had no detrimental effects on mouse homeostasis.

### PSOn supplementation does not impact whole body weight in mice fed a high-fat diet

**Figure 1:**
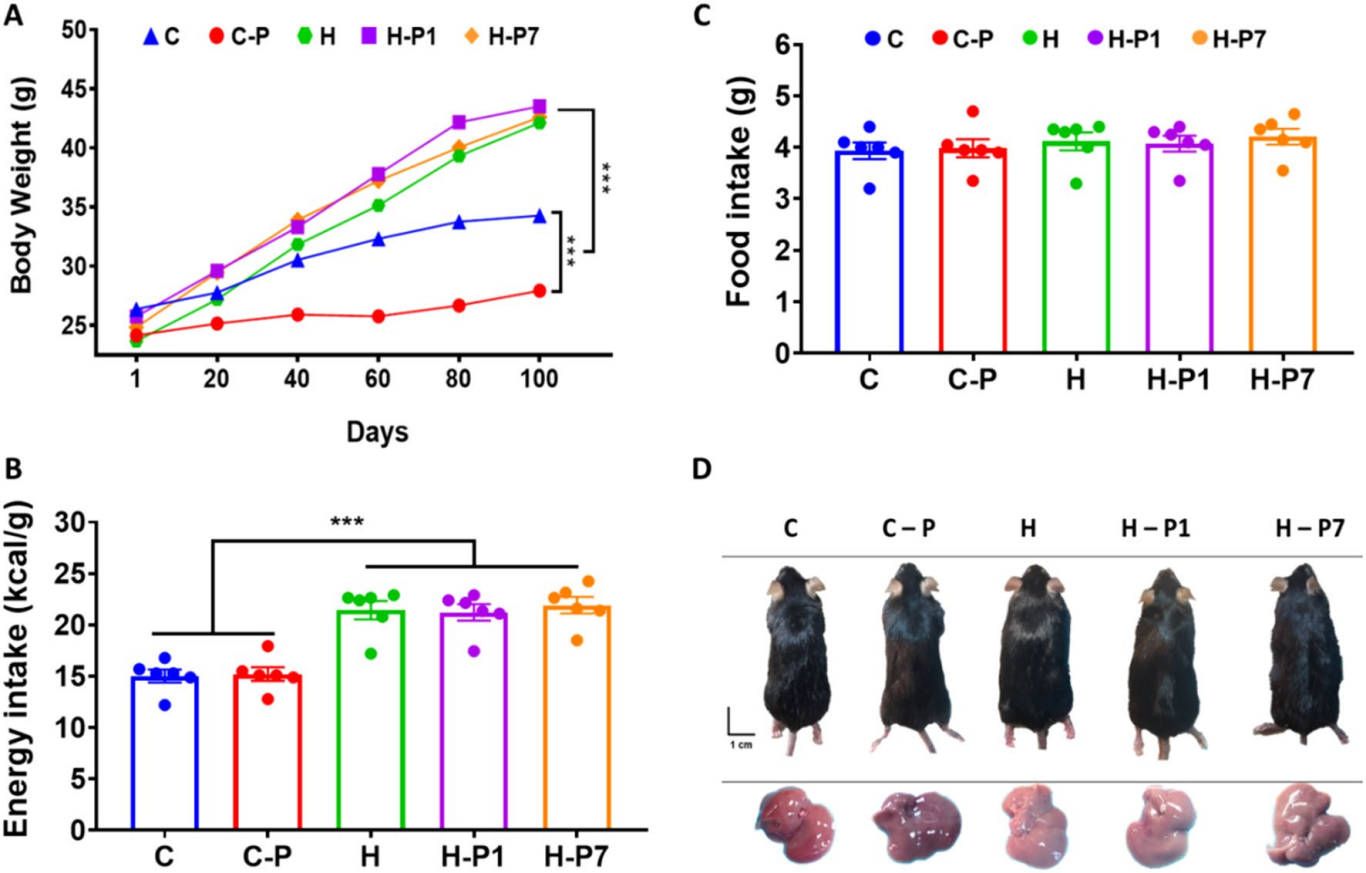
Overall effect of the HFD-fed mice supplemented with PSOn. (**A**) The whole body weight of the different mouse groups is shown during the diet time-course administration; lines represent the mean ± standard error of the mean (SEM) from each group (***p < 0.0001 between the indicated groups). The total calorie (**B**) and food amount (**C**) intake are plotted for each of the dietary groups, where the bars indicate the mean (SEM) from each group (***p < 0.01). (**D**) Representative photographs of mice and their corresponding liver organs for each dietary group at the end of the treatment.

In previous reports, PSO supplementation has been related to prevention of diet-induced obesity and other beneficial effects. As high-fat diet-mediated fatty liver disease is tightly associated with obesity, we hypothesized that PSOn could prevent or reverse obesity and triglyceride hepatic deposition in our mice. Interestingly, as shown in Fig. 1A, we found that control mice supplemented with PSOn (C-P) gained significantly less body weight compared to chow-fed mice (3.48 g vs. 8.04 g, respectively, *p* < 0.001); on the contrary, and as expected, the high-fat diet (HFD) significantly increased mouse body weight compared to the chow or chow-PSOn group (*p* < 0.001); unexpectedly, our HFD-fed mice supplemented with PSOn at week 1 (H-P1) or at week 7 (H-P7) did not lose any body weight compared to the HFD-fed group (Fig. 1A). Interestingly, the higher body weight gain observed in the HFD group was not due to differences in food consumption, since all of the groups showed similar food intake behaviour, but to an increase in energy intake (Fig. 1B,C). In addition, and as expected, the HFD-fed mice had an obese body phenotype and their livers showed signs of steatosis when compared to control mice (Fig. 1D). With regard to the lipid profile, we did not observe any favourable improvement. However, AST and ALT enzymes were slightly lower after PSOn supplementation in both the H-P1 and H-P7 mouse groups compared to the HFD group; albumin was also significantly augmented in the H-P7 group, indicating that PSOn might improve hepatic damage induced by HFD (Table 1).

**Table 1:** Biochemical characteristics.

Parameter	C	C-P	HFD	H-P1	H-P7
ALT (U/L)	21.0 ± 3.1	37.0 ± 5.9	31.9 ± 6.5	33.8 ± 5.1	25.9 ± 2.7
AST (U/L)	105.1 ± 33.8	82.9 ± 11.4	94.2 ± 8.3	84.0 ± 10.3	80.6 ± 8.1
Albumin (g/L)	1.3 ± 0.01	1.7 ± 0.2	1.5 ± 0.1	1.6 ± 0.05	2.8 ± 0.1***
Urea (mg/dL)	34.8 ± 1.4	28.4 ± 2.03	30.0 ± 1.2	28.1 ± 1.3	29.1 ± 0.5
Glucose (mg/dL)	281.4 ± 24.2	263.4 ± 26.4	340.9 ± 23.4	330.2 ± 11.5	260.6 ± 10.7*
Triglycerides (mg/dL)	70.7 ± 6.5	60.4 ± 4.2	41.7 ± 4.02	55.2 ± 3.9	56.1 ± 3.9*
Cholesterol (mg/dL)	76.5 ± 5.09	64.4 ± 7.4	111.8 ± 9.8	117.6 ± 4.6	126.9 ± 7.1
HDL (mg/dL)	96.1 ± 5.6	72.1 ± 8.5	126.5 ± 8.7	141.0 ± 7.2	136.8 ± 4.9
LDL (mg/dL)	14.3 ± 1.5	9.4 ± 0.7	22.1 ± 2.7	25.1 ± 1.9	26.6 ± 2.2

*AST* aspartate amino-transferase, *ALT* alanine amino-transferase, *HDL* high-density lipoprotein, *LDL* low-density lipoprotein. T test *p < 0.05 vs. HFD; ANOVA ***p < 0.001 vs. HFD & H-P1.

### PSOn administration does not impact body composition

**Figure 2:**
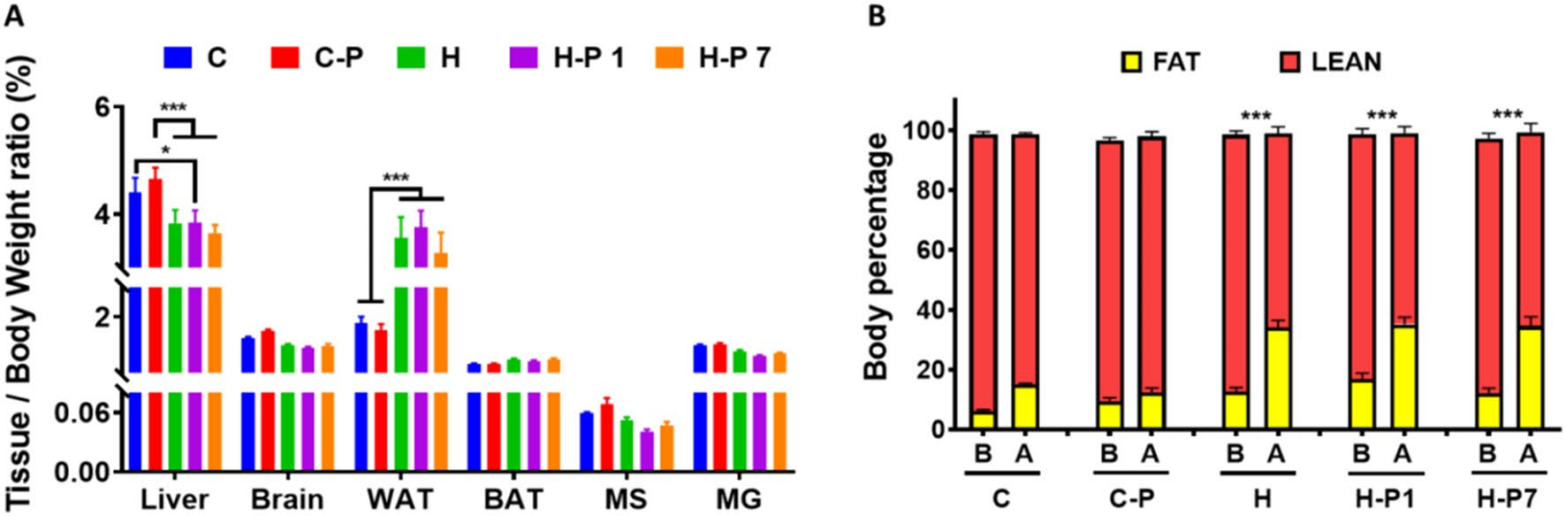
PSOn supplementation does not have an impact on whole body composition. (**A**) The tissue/body weight ratio is shown as a percentage of each of the indicated organs (WAT, white adipose tissue; BAT, brown adipose tissue; MS, soleus muscle; GM, gastrocnemius muscle); bars represent the mean ± SEM for each mouse group. (**B**) Body composition analysis by magnetic resonance imaging (MRI) indicates the difference between fat and lean mass in mice before (**B**) and after (**A**) the diet and supplementation time (*p < 0.05 & ***p < 0.01).

We then decided to assess the relationship between whole mouse body weight and organ weight in order to determine whether mice suffer from any gross organ size disturbance after PSOn treatment. As observed in Fig. 2A, mice under HFD had a significantly lower liver/body weight ratio, which can be explained by the higher body mass when compared to controls, whereas white adipose tissue mass showed the opposite ratio (*p* < 0.001), surely due to the fat mass gain at the expense of muscle mass loss, compared to the control mouse groups (Fig. 2A). To further corroborate these data, we performed magnetic resonance imaging to evaluate whether those mice were different with respect to body fat or lean mass. Here, we confirmed that HFD-fed mice gained significantly more fat mass on average, 21.4 g (*p* < 0.001), compared to their basal levels and the control groups, while the muscle mass was proportionally lower (Fig. 2B), and PSOn treatment did not modify the loss of lean mass. These results indicate that PSOn has no effect on body composition in mice fed a HFD.

### PSOn supplementation reduces glucose intolerance and insulin resistance in HFD-fed mice

**Figure 3:**
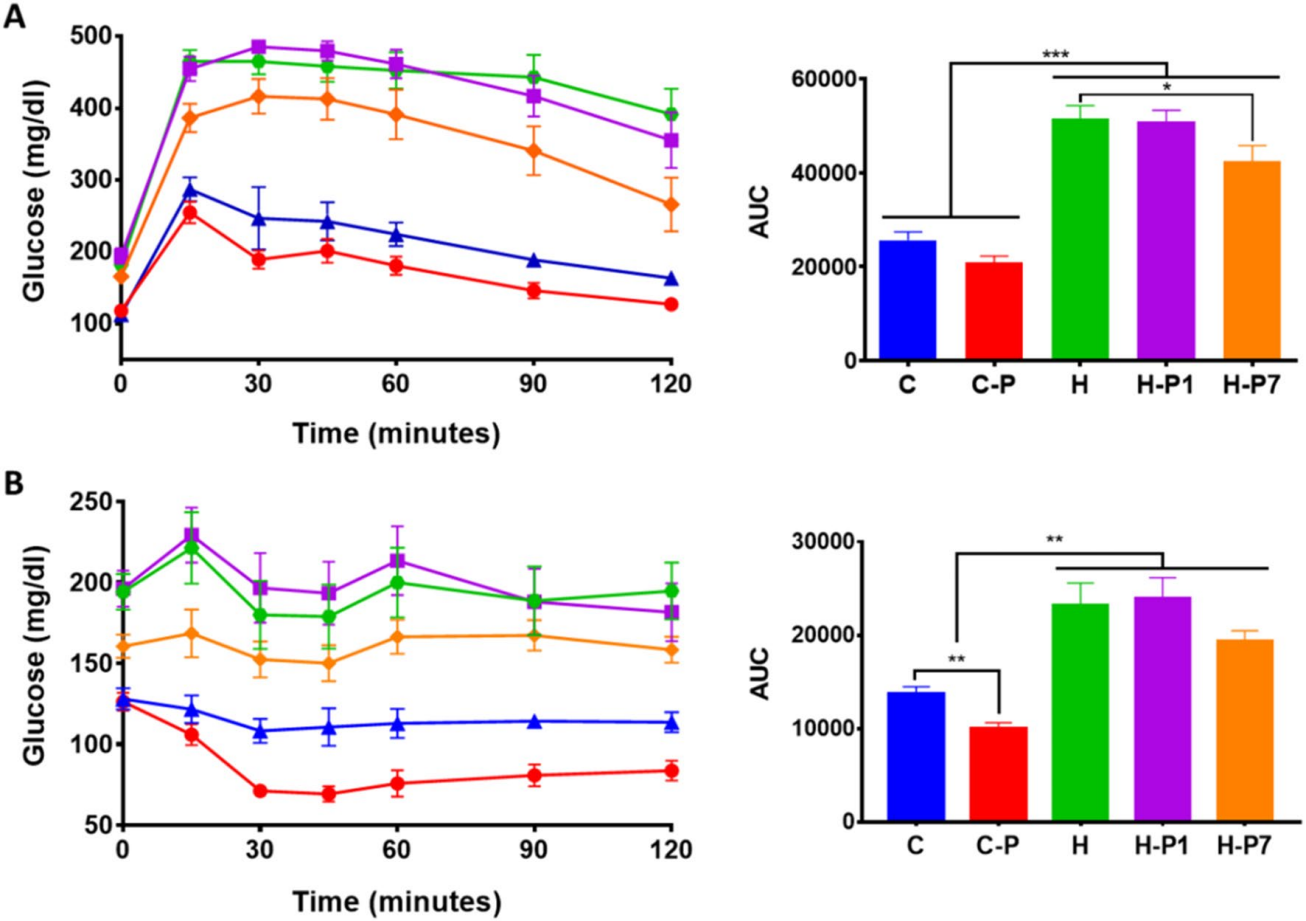
PSOn treatment reduces glucose intolerance and insulin resistance in HFD-fed mice. The time-course blood glucose levels are plotted for each group after the ipGTT (**A**) or ipITT (**B**) as described in the “Materials and methods”. The area under the curve (AUC) was determined and is shown on the right panel for each test. The data are shown as the mean ± SEM. *p < 0.05 by Student’s t test vs. HFD; **p < 0.01 by Student’s t test C vs. C-P; ANOVA **p < 0.01 C and C-P vs. H, H-P1 and H-P7; ANOVA ***p < 0.001 C and C-P vs. H, H-P1 and H-P7.

As PSO has been reported to play a role in insulin and glucose regulation, we sought to investigate whether the supplementation of PSOn modifies the glucose intolerance induced by the HFD. Thus, we performed intraperitoneal glucose and insulin tolerance tests. As expected, control mice efficiently regulate their glucose levels along the time interval, reaching basal levels at the end of the experiment (Fig. 3A). In contrast, HFD-fed mice showed glucose intolerance (*p* < 0.0001 vs. C or C-P groups) and remained hyperglycaemic after 120 min (Fig. 3A, left panel). The area under the curve plots showed that PSOn supplementation in the H-P1 group did not improve the glucose intolerance induced by HFD; however, when administered at week 7 (H-P7), PSOn significantly (*p* < 0.05) diminished blood glucose levels (Fig. 3A, right panel). Interestingly, the insulin tolerance test showed a significant drop in glucose levels in the C-P mouse group when compared with the control group (Fig. 3B, *p* < 0.01); we observed that HP1 has no effect on insulin sensitivity, whereas the glucose levels showed a striking decline in the H-P7 group, although they did not reach a significant difference when compared to the HFD group (*p* < 0.17) (Fig. 3B, right panel). Based on these observations, we conclude that PSOn is able to improve glucose and insulin metabolism in control and HFD-fed mice.

### PSOn supplementation increases energy expenditure without modifying substrate utilization

**Figure 4:**
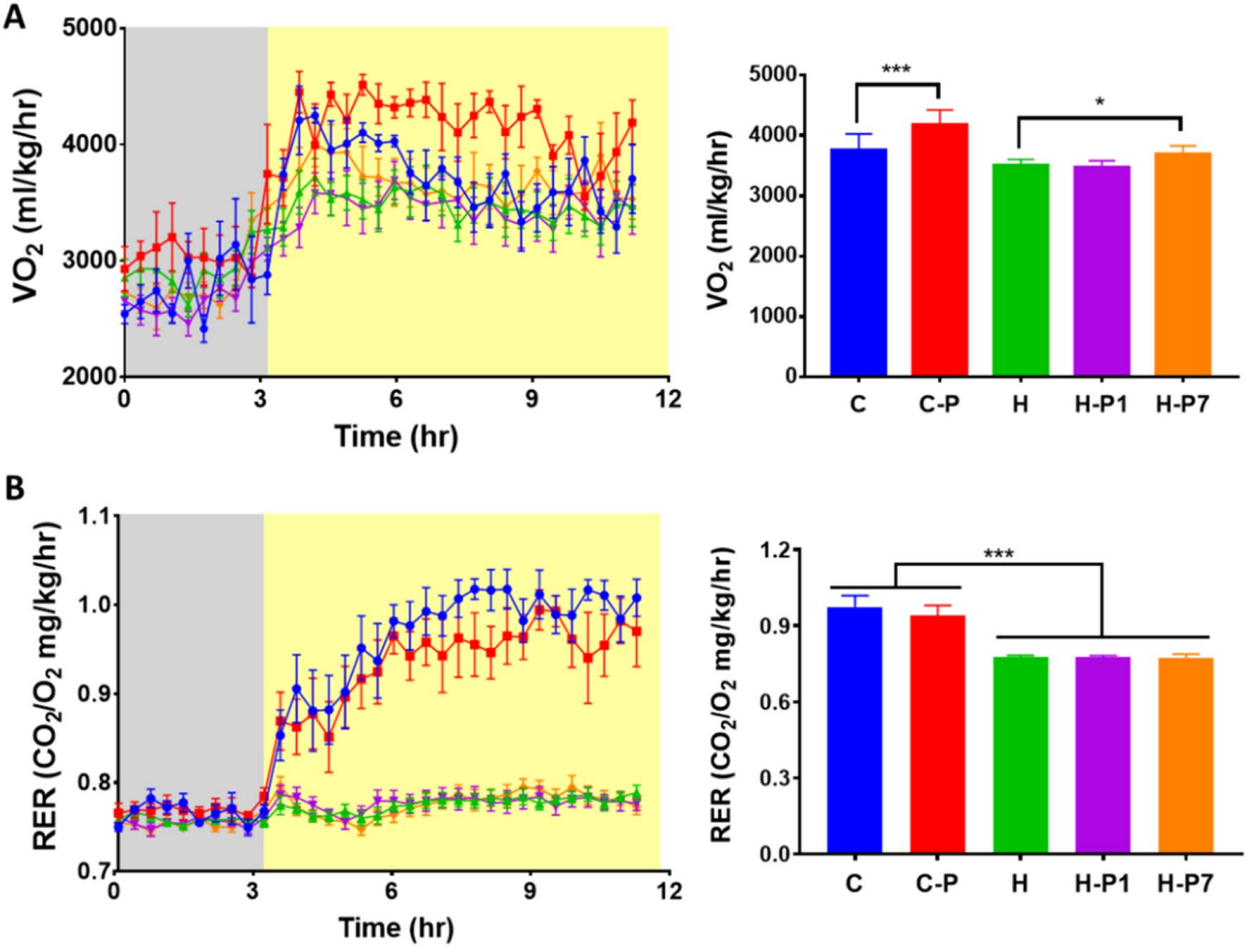
PSOn increased energy expenditure without modification of substrate. Mice from each diet group were subjected to the indirect calorimetric assay in fasting (grey areas in **A** & **B**) and feeding (yellow areas in **A** & **B**) conditions. Oxygen (VO_2_) consumption (**A**) and the respiratory exchange ratio (RER) (**B**) were measured at the indicated time points. The quantitation for both parameters is shown after the feeding condition on the right panels. Data represent the mean ± SEM for each group; *p < 0.05; ***p < 0.001.

To evaluate whether PSOn modifies energy expenditure or substrate utilization in HFD-fed mice, we conducted an indirect calorimetric assay. Notably, we observed that PSOn significantly increased oxygen consumption by 1.2-fold in mice fed the control diet (*p* < 0.0001). As expected, HFD-fed mice significantly reduced their oxygen consumption rate by a 0.9-fold change (*p* < 0.001) compared to control mice (Fig. 4A, right plot). Interestingly, when HFD-fed mice were supplemented with PSOn since the beginning of the diet regime, oxygen consumption did not improve and, instead, it remained the same as for mice from the high-fat diet group (3,465.5 vs. 3,494.5, ml/kg/h, *p* < 0.99) (Fig. 4A). However, HFD-fed mice supplemented in week 7 (H-P7) successfully and significantly increased their oxygen consumption rate almost to the level of the chow control mouse group (3,681 vs. 3,748, ml/kg/h, *p* < 0.99) (Fig. 4A). With regard to the respiratory exchange ratio (RER), as can be observed in Fig. 4B, all of the groups had a RER of approximately 0.75 under fasted conditions, indicating that fatty acids are the main substrate being oxidized. As expected, the control groups increased their RER to 1 in re-fed conditions, indicating the switch to glucose as the substrate being oxidized. However, the HFD-fed mice continued to have a RER of 0.75, indicating the presence of metabolic inflexibility. PSOn supplementation, either from the beginning (H-P1) or in week 7 (H-P7), did not improve this metabolic inflexibility (Fig. 4B). Altogether, these results indicate that PSO increases energy expenditure without modifying substrate utilization.

### PSOn supplementation reverses high-fat diet-induced hepatic steatosis

**Figure 5:**
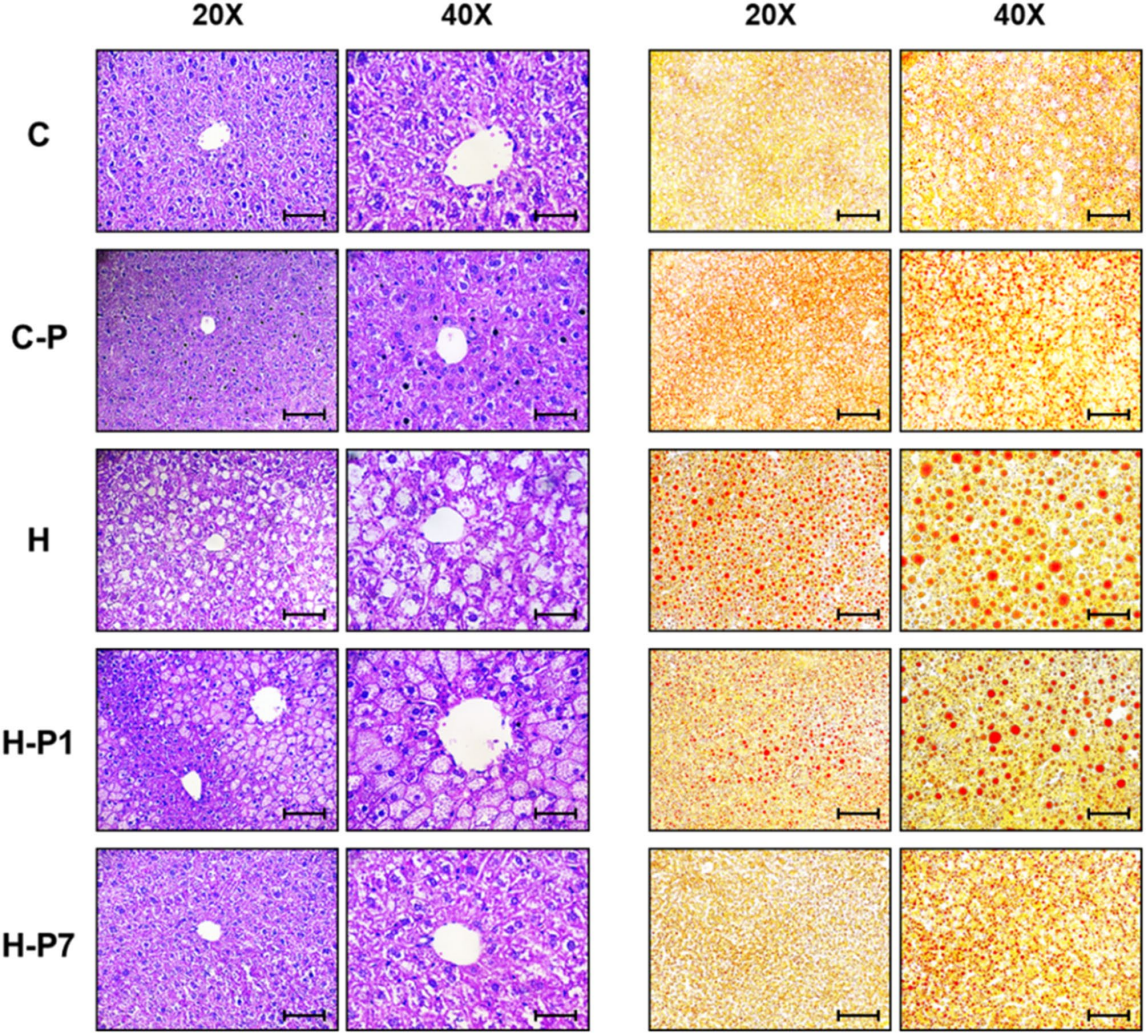
PSOn supplementation reverses HFD-induced hepatic steatosis. Representative micrographs of liver histological sections stained with haematoxylin–eosin (H&E, left panel) and oil red O (ORO, right panel) from the indicated mouse groups. The objective magnification is indicated at the top of each panel. Scale bars in ×20 and ×40 are 100 μM and 50 μM, respectively.

Given that hepatic steatosis, glucose intolerance and insulin resistance are common complications during obesity and that our data indicate that PSOn treatment effectively improved glucose and insulin levels in HFD-fed mice, we decided to evaluate if the hepatic steatosis could also be improved. To this end, we performed histological oil red O (ORO) staining on mouse liver tissue sections. As observed in Fig. 5, HFD-fed mice clearly showed lipid droplet accumulation in the liver parenchyma compared to control diet-fed mice. This pattern was also observed in the H-P1 group, although to a lesser extent. In contrast, mice treated with PSOn at week 7 (H-P7) clearly displayed a reversion of this phenotype at the end of the experiment (Fig. 5). Taken together, these data suggest that PSOn does not cause tissue damage and reverses but does not prevent liver lipid droplet accumulation.

### PSOn increases fatty acid oxidation in hepatocytes

**Figure 6:**
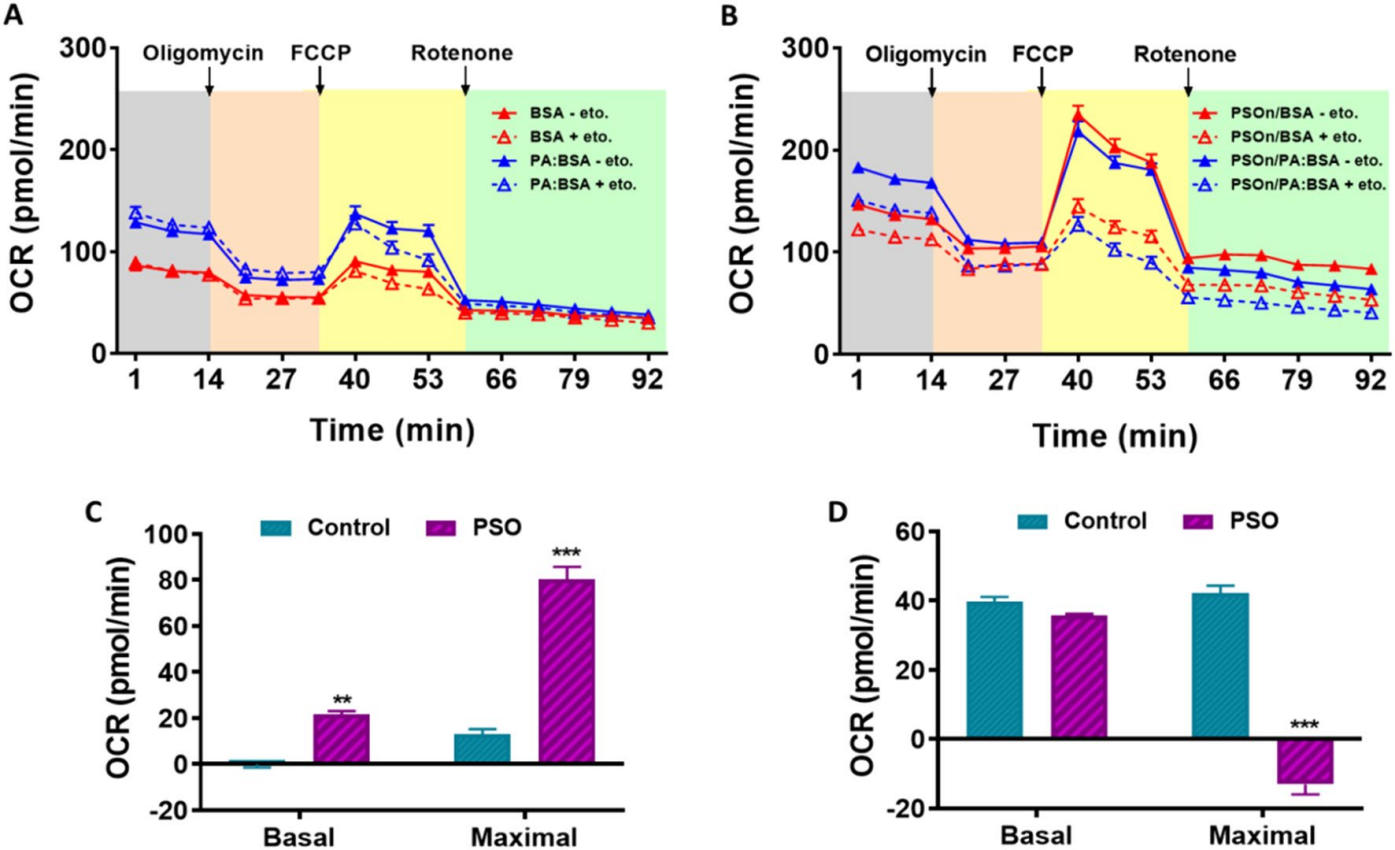
PSOn increases fatty acid oxidation in hepatocytes. Assessment of the mitochondrial function based on the oxygen consumption rate (OCR) in a time-course experiment is indicated for control (**A**) or stimulated (**B**) conditions. The endogenous (BSA alone) (**C**) and exogenous (Palmitate:BSA) (**D**) fatty acid oxidation was evaluated in basal or maximal conditions for control or PSOn-supplemented cells in the presence (** +**) or absence (**−**) of etomoxir (eto) and plotted as the OCR. Bars indicate the mean ± SEM for each group (***p < 0.01; FCCP, carbonyl cyanide-p-trifluoromethoxyphenylhydrazone).

As PSOn treatment reduced hepatic steatosis, we evaluated mitochondrial function and fatty acid oxidation in primary hepatocytes to determine whether PSOn exerts a direct effect on these cells. First, we tested the safety of PSOn exposure using different cell lines in vitro. We measured cell viability and whole integrity by means of dose–response and time-course experiments in myoblasts (C2C12 cells), fibroblasts (3T3-L1, cells) and mouse brain primary astrocytes. Interestingly, increasing concentrations of PSOn did not modify cell viability or whole cell morphology in any of the cells tested (Supplementary Fig. 3A,B). Therefore, we incubated mouse primary hepatocytes with 1 mg/ml PSOn for 3 or 18 h and performed a mitochondrial stress test in order to determine the effect of PSOn on mitochondrial function using an extracellular flux analyser (SeahorseXFe96 analyser, Agilent Technologies). Unexpectedly, the basal oxygen consumption rate (OCR) and key parameters of mitochondrial function such as ATP production, proton leak, maximal respiration, or spare respiratory capacity were not different between hepatocytes incubated with vehicle or PSOn at the indicated times (Supplementary Fig. 4), indicating that PSOn does not modify mitochondrial function. Thus, we then evaluated the oxidation of exogenous and endogenous fatty acids to determine whether the decrease in hepatic lipid droplets was specifically due to an increase in fatty acid oxidation. To test this hypothesis, we performed a mitochondrial stress test in primary hepatocytes incubated in the presence or absence of PSOn (1 mg/ml) for 3 h in a medium containing BSA to measure endogenous FA oxidation or with palmitate-BSA to measure exogenous FA oxidation, with or without the carnitine-palmitoyltransferase-1A (CPT1A) inhibitor, etomoxir. Interestingly, PSOn increased basal respiration and maximal respiration when glucose was limited (Fig. 6A,B). Furthermore, PSOn increased the endogenous FA oxidation capacity of hepatocytes in both basal and maximal respiration conditions (*p* < 0.01) (Fig. 6C,D). Altogether, these data indicate that PSOn significantly increases fatty acid oxidation capacity in hepatocytes and thus helps to reverse the hepatic steatosis induced by a HFD.

### PSOn increases the antioxidant- and lipid metabolism-related gene expression

**Figure 7:**
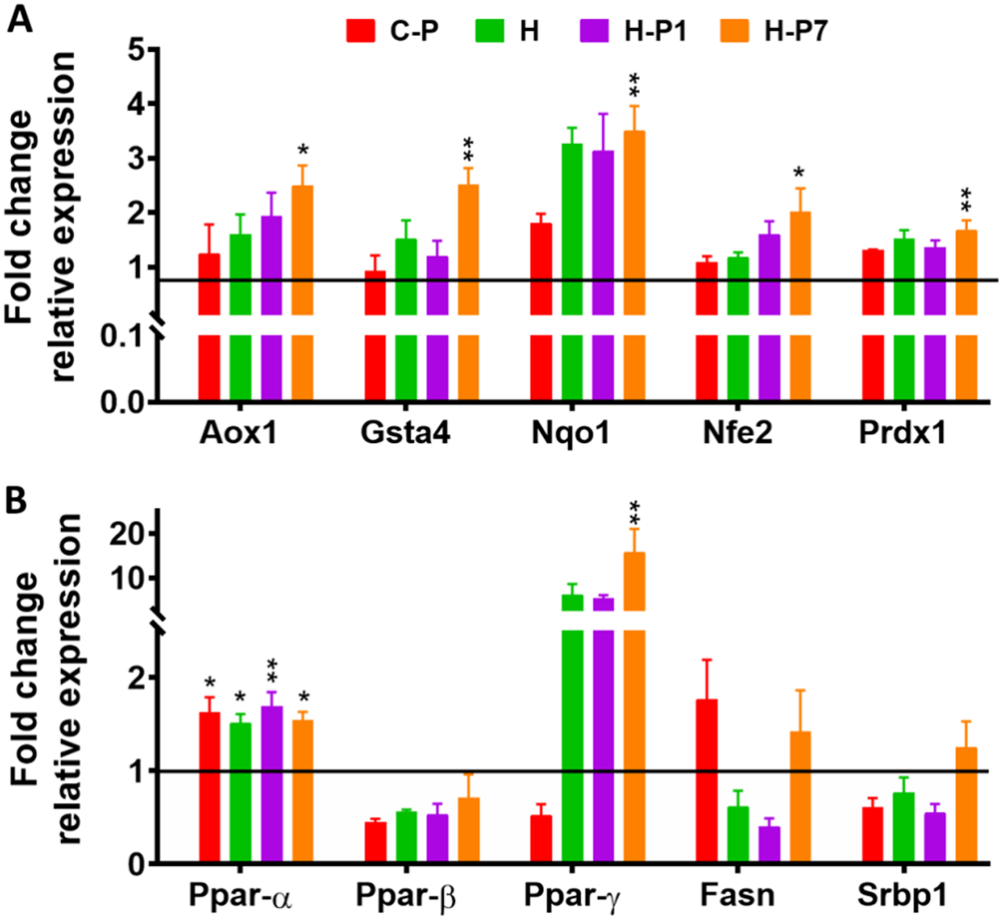
PSOn increases the antioxidant- and lipid metabolism-related gene expression. Determination of transcripts was made by total RNA extraction from liver tissue and quantitated by qPCR. Representative data are shown. The bars represent the mean ± SEM of the relative expression for each gene and the indicated mouse groups (Aox1, aldehyde oxidase; Gsta4, glutathione S-transferase 4; Nqo1, NADPH-quinone dehydrogenase 1; Nfe2, nuclear factor erythroid 2; Prdx1, peroxiredoxin 1). *p < 0.05; **p < 0.001 vs. HFD group.

As PSO has been reported to have antioxidant properties^11^, we decided to measure the expression of several antioxidant transcripts in the liver of the different mouse groups to evaluate whether PSOn preserved the antioxidant properties. As we expected, the PCR experiments showed that PSOn supplementation increased the expression of several antioxidant genes (aldehyde oxidase 1 (Aox1), glutathione S-transferase A4 (Gst4), NAD(P)H quinone dehydrogenase 1 (Nqo1), nuclear factor erythroid 2-like 2 (Nrf2), and peroxiredoxin 1 (Prdx1), among others), when administered since the beginning of the diet supplementation (H-P1); however, the increase was larger in the livers of mice supplemented at week 7 (H-P7), consistent with the rest of our metabolic findings (Fig. 7A). In addition, we also observed that lipid metabolism-associated transcripts such as peroxisome proliferator activated receptor-alpha (Pparα), Pparβ or Pparγ, as well as fatty acid synthase (Fasn), and sterol regulatory element binding transcription factor (Srbp1), were significantly up regulated in the liver of high-fat diet-fed mice (Fig. 7B). These results indicate that PSOn increased the expression of antioxidant- and lipid metabolism-related genes in the livers of mice fed a high-fat diet.

## Discussion

Punicic acid, also known as omega-5 (ω-5), is a conjugated fatty acid that exerts health benefits in a variety of chronic pathologies such as diabetes, inflammation, hyperlipidaemia, and nephrotoxicity, among others^22^. In this study, we report that HFD-induced obese mice successfully improve their metabolic disturbances when treated with PSOn. In a previous report, PSO supplementation for 14 weeks in mice fed a high-fat diet significantly reduced their body weight gain^9^. In contrast, we observed that only the C-P mice reduced this parameter when compared to a control mouse group, while this effect was not observed in mice under HFD supplemented with PSOn, neither from the beginning or when supplemented at week 7^6,9^. This discrepancy can partially been explained by differences in food consumption observed in both studies and by the strain of mice used. It is possible that the chow diet/PSO mix experimental strategy might have altered the taste of the food for mice while ingesting it, hence inducing a direct impact on the body weight gain. To overcome this issue, we decided to directly administer the PSOn by oral gavage, which in turn had no effect on food consumption, since all of our mouse groups showed no differences in food intake. However, mice did have significant differences in energy intake, which easily explains the body weight-gain differences observed in the HFD-fed mice. In line with this observation are the results of some studies indicating that pomegranate seed oil supplementation in mice induces weight gain despite a similar energy intake^6,9^. Today, a study conducted in Balb/c mice clearly showed that pomegranate peel extract did not modify body weight gain under a HFD regime^23^. The lowering effect on the chow-PSOn group body weight might also be related to the gut microbiota or the energy expenditure capacity, as discussed later. The registered body weight in our mice, however, is consistent with the observed results of body composition analysis, which indicates that HFD fed-mice gained the same amount of fat as those with PSOn supplementation at the expense of lean mass.

On the other hand, it is well known that one of the most important metabolic disturbances in obese mice or humans is the glucose and insulin levels and their associated endocrine responses at the peripheral and central levels^24–27^. According to the intraperitoneal-glucose and insulin tolerance test (ipGTT and ipITT, respectively) results, PSOn induced an improvement in glucose tolerance and insulin sensitivity in control mice and mice supplemented at week 7 (H-P7). These results are similar to those reported in a HFD-induced obese CD-1 mouse model^9^. Accordingly, the insulin sensitivity of the H-P7 mouse group showed a trend toward being reduced, in agreement with the data reported by Vroegrijk et al*.*, in an obese mouse model^6^. It has been suggested that the punicic acid hypoglycaemic effects are mediated by PPARγ, which might inactivate the pro-inflammatory pathway mediated by nuclear factor-kappa-B (NF-κB) and tumour necrosis factor-alpha (TNF-α)^28^.

A controversial result is the fact that PSOn did not successfully prevent the fatty liver phenotype when administered since the beginning of the HFD, but only when it was supplemented at week 7. We propose that this effect was due to the omega-5 metabolism of the host that, administered along with the high-fat diet, is processed towards the fatty acid metabolic pathways in order to generate new fatty acids that might accumulate within the liver parenchyma^29–31^.

Since the obese mice from the H-P7 group had significantly reduced liver steatosis along with the glucose and insulin responses, we thought this could be related to a total energy expenditure-related issue or that their liver parenchyma was improving its physiological activity.

In terms of energy expenditure, the observed macronutrient contribution to the respiratory exchange ratio (RER) indicates that independently of the PSOn supplementation, the H-P1 and H-P7 mouse groups are metabolically inflexible, due to continuous fat utilization after the re-feeding period, when compared to the chow diet groups (see Fig. 4B). These results showed an improved liver steatosis in the H-P7 group, as shown by the oil red O (ORO) and haematoxylin and eosin (H&E) staining, which strongly suggests that the liver parenchyma was improving its functional physiological activity. In line with this hypothesis, our data for the oxygen consumption rate (OCR) indicate that PSOn effectively increased the endogenous fatty acid oxidation during basal and maximal respiratory capacity in primary hepatocytes (Fig. 6C). To our knowledge, these data are the first regarding fatty acid oxidation in the presence of PSOn in primary hepatocytes; therefore, no further data are available to properly compare our results. However, metabolic data derived from different conjugated linoleic acids (CLnA), such as omega-3, have been reported to actively induce the oxygen consumption rate in skeletal muscle cells by means of inducing an increased amount of mitochondrial content^32^.

During obesity, the insulin-mediated intracellular pathway is disturbed by, among others, the action of inflammatory cytokines or by lipotoxicity, both of which might promote the increase in free radicals and endoplasmic reticulum stress, which interfere with the insulin receptor phosphorylation mechanisms^26^. It is plausible that an important contribution to the results we observed in our study more likely comes from the potent antioxidant activity exerted by the CLnAs, as reported previously^33^. Specifically, it has been shown that malondialdehyde (MDA) or thiobarbituric acid-reactive substances (TBARS) levels, both indicators of lipid peroxidation, were significantly regulated in tissue or blood-derived samples from CLnA-supplemented murine models, under basal^34^ or pathological conditions^14,35,36^. The CLnA-mediated effects seem to be related to the augmented expression of antioxidant-encoded gene enzymes such as catalase (CAT), glutathione peroxidase (GPx), superoxide dismutase (SOD), and others^34^. Our results indicate that the transcript expression of the genes encoding some antioxidant-related proteins, such as Aox1, Nfe2, and one of its targets, Nqo1, as well as Prdx1 transcripts, was also significantly increased in mice supplemented with PSOn, suggesting the antioxidant protective role of this nanoemulsion at physiological levels. Furthermore, we have found that, at least in our model, PSOn induces fatty acid oxidation in hepatocytes, thus contributing to lowering the accumulation of triglycerides within the liver under obesogenic conditions. Additional clinical assays will be necessary in order to demonstrate these beneficial effects in human subjects under basal or pathological conditions.

## Materials and methods

### Animal care and PSOn supplementation maintenance

The Animal Care and Ethics Committee of the INCMNSZ approved all of the procedures related to animal handling and experimentation. We confirm that all the experiments performed in this study are in accordance with the Mexican Regulations (NOM-062-ZOO-1999). Eight-week-old male C57BL/6N (Charles River) mice were housed in environmentally enriched cages (4 mice/cage) in a room with a controlled temperature of 21 ± 3 °C and illuminated with 12/12 h of light/dark per day. The animals received a standard diet and water ad libitum for 7 days before the beginning of the treatments. The mice were randomly divided into 5 dietary intervention groups: 5 mice with control (C) diet (D12450K, Research Diets), 10 mice with control diet supplemented with 3.5 mg/g PSOn (CP), 10 mice with high-fat diet (H) (D12492, Research Diets), 10 mice with high-fat diet supplemented with 3.5 mg/g PSOn diet from week 1 (H-P1) and 10 mice with high-fat diet supplemented with 3.5 mg/g PSOn beginning at week 7 (H-P7). Both diets and water were administered ad libitum. PSOn (GranaGard) was administered every other day by oral gavage for 15 (HP1) or 8 (HP-7) weeks. This dose was chosen based on previous studies that report doses between 1 and 5%^6,9,37^. The body weight and food intake were quantified every 4 days. On the last day of the treatments, the mice were anaesthetized with an overdose of pentobarbital, blood was collected by cardiac puncture, and some organs were removed for later study.

### Biochemical and cellular peripheral blood measurements

Peripheral blood was collected in EDTA-treated tubes by cardiac puncture before euthanasia and under deep anaesthesia. Plasma was obtained from blood by centrifugation at 10,800×*g* for 4 min and stored at − 70 °C until use. Alanine aminotransferase (ALT), aspartate aminotransferase (AST), albumin, glucose, urea, triglycerides, and cholesterol were determined by colourimetric assays using the biochemical analyser COBAS c111 (Roche Diagnostics, Indianapolis, IN).

### Intraperitoneal glucose and insulin tolerance tests

Intraperitoneal glucose tolerance tests (ipGTT) and insulin tolerance tests (ipITT) were performed on week 15 and 16, respectively. In brief, mice fasted for 6 h before each test. The IpGTT was initiated by an intraperitoneal injection of 2 g/kg of glucose and the ipITT by intraperitoneal injection of 0.5 UI/kg of insulin. Glucose was measured in blood from the tail vein at 0, 15, 30, 45, 60, 90 and 120 min after the injection using a portable FreeStyle glucometer (Abbott).

### Whole body composition and energy expenditure measurements

Body composition (lean and fat mass) was evaluated at week 18 using magnetic resonance imaging (EchoMRI; Echo Medical Systems, Houston, TX, USA). Energy expenditure and substrate utilization were evaluated at week 14 by indirect calorimetry using the Oxymax CLAMS system (Comprehensive Laboratory Animal Monitoring System, Columbus, OH). Briefly, mice were acclimatized for 24 h in the CLAMS cages before data collection. Mice were food deprived for 6 h for fasting recordings and then fed their corresponding diets for the next 18 h. Oxygen (O_2_) consumption and carbon dioxide (CO_2_) production were continuously measured throughout the test. The respiratory exchange ratio (RER) was determined as the volume of CO_2_ exhaled (VCO_2_, ml·kg^−1^·h^−1^) divided by the volume of O_2_ used (VO_2_, ml·kg^−1^·h^−1^).

### H&E and ORO histological staining

Liver or kidney tissue samples were fixed with 4% (w/v) paraformaldehyde in PBS and embedded in paraffin blocks. Then, 5-µm sections were stained with haematoxylin and eosin. For oil red O stain, 8 µm frozen OCT-embedded liver tissue sections were obtained with a pedestal cryostat. Stained slides were imaged under a Leica DM750 microscope (Leica, Wetzlar, Germany) at 20 and 40X magnification.

### Quantitative real-time PCR

Total RNA was obtained from 100 mg of liver tissue using TRIzol reagent (Invitrogen) followed by quantification using a Nano Drop spectrophotometer (Nano Drop Technologies). The 260/280 nm wavelength ratio > 1.5 was used as a purity indicator. Then, 1 μg of total RNA was reverse transcribed according to the Transcriptor First Strand cDNA Synthesis Kit guidelines from Roche (Roche, Mannheim Germany). PCR amplification was performed in a Roche Light Cycler 480 II using SYBR Green (QuantiTect PCR Kit, Qiagen). The PCR primer sequences are shown in the supplementary Table 2. The PCR conditions were as follows: pre-incubation at 95 °C for 10 min, followed by 45 cycles of denaturing at 95 °C for 10 s, annealing at 60 °C for 30 s and extension at 72 °C for 10 s, finally cooling at 40 °C for 30 s. For gene expression, the levels were relative to the values of 18S expression, and the results were expressed as fold changes of threshold cycle (Ct), calculated with the delta-Ct method (2^−ΔΔCt^).

### Primary hepatocyte cell culture and mitochondrial stress test

Primary hepatocytes were collected by in situ perfusion of the liver according to the method of Berry MN and Friend DS^38^, which consists of cannulating and exsanguinating the liver in vivo*,* followed by a continuous perfusion with collagenase. The livers were then isolated and placed in Hanks balanced salt solution (HBSS) to disaggregate the tissue. The cell suspension was then filtered through a 70-μm mesh. The filtered hepatocytes were washed twice with HBSS and finally re-suspended in M199 medium supplemented with 10% foetal bovine serum (FBS), antibiotic, 0.1% bovine serum albumin (BSA), 1 nM insulin, 100 nM dexamethasone and 100 nM triiodothyronine (T3). Cells were then seeded into an XFe96 microplate at a density of 4,000 cells/well. The medium was changed after 4 h to remove unattached cells. The next day, cells were incubated with PSOn (1 mg/ml) for 3 h, and then the mitochondrial function was evaluated, as well as the endogenous and exogenous fatty acid oxidation using a mitochondrial stress test in an XFe96 Extracellular Flux Analyser (Agilent Technologies). Briefly, cells were washed and incubated for 1 h in a non-CO_2_ incubator with XF basal medium supplemented with 10 mM glucose, 1 mM pyruvate and 2 mM glutamine. During the experiment, 2 μM oligomycin, 1 μM carbonyl cyanide-p-trifluoromethoxy phenyl-hydrazone (FCCP) and 1 μM rotenone/antimycin A were injected sequentially, and three measurements were performed in basal conditions and after the addition of each compound. The oxygen consumption ratio (OCR) measurements were obtained and analysed according to the manufacturer’s recommendations. To evaluate the endogenous fatty acid (FA) oxidation, the mitochondrial stress test was performed in basal medium containing BSA (170 μM), while exogenous FA oxidation was determined in the presence of a palmitate/BSA (100 μM/170 μM) mixture, both in the presence or absence of the CPT1A-inhibitor etomoxir.

### Statistical analysis

Unless otherwise indicated, all the results are presented as the mean ± standard error of the mean (SEM). The differences among groups were analysed with one-way analysis of variance (ANOVA) followed by Tukey’s or Bonferroni tests, using Graph Pad PRISM (v7.0). Differences among means were compared at a level of significance of p < 0.05. Each experiment was performed at least in triplicate.

## Supplementary information

Supplementary information
