# Supplementary material for: Punica granatum L.-derived omega-5 nanoemulsion improves hepatic steatosis in mice fed a high fat diet by increasing fatty acid utilization in hepatocytes
Source: Sci Rep. 2020 Sep 17;10:15229. doi: 10.1038/s41598-020-71878-y (PMC7499212; doi:10.1038/s41598-020-71878-y)
Supplement: Supplementary file 1 — Supplementary information [file 41598_2020_71878_MOESM1_ESM.pdf]

Supplementary material

Punica granatum L.-derived omega-5 nanoemulsion improves hepatic steatosis in mice fed a high fat diet by increasing fatty acid utilization in hepatocytes

Zamora-López K.

Noriega L.G.

Estanes-Hernández A.

Escalona-Nández I.

Tobón-Cornejo S.

Tovar A. R.

Barbero-Becerra V.

Pérez-Monter C.

1. Supplementary Methods

S1. Animal care and PSOn supplementation maintenance

The Animal Care and Ethics Committee of the INCMNSZ approved all of the procedures related to animal handling and experimentation. Eight-week-old male C57BL/6N mice were housed in environmentally enriched cages. Food (a standard rodent chow diet) and water were provided *ad libitum*. The circadian rhythm was controlled with a 12/12-h light/dark cycle. For the acute protocol, mice were divided into a control group and 1 mg/kg, 2 mg/kg and 4 mg/g PSOn groups (n=5 each). The PSOn was administered in mice once by oral gavage, and mice were followed up to 15 days. We also sought to test the long-term effect of PSOn in an every other day administration basis (chronic protocol); here, mice were divided into a control or PSOn group (n=5 each) and the same oral gavage strategy was used to administer three different doses (8.7 mg/g, 26 mg/g and 35 mg/g of PSOn body weight); mice were followed up to 28 days. Body weight and food consumption were monitored every 3 days. At the end of both protocols, mice were euthanized by pentobarbital injection and decapitated; peripheral blood, liver and kidneys were collected for further analysis. Tissues from both protocols were processed for H&E staining.

S2. Biochemical and cellular peripheral blood measurements

Plasma was obtained from peripheral blood and used to determine the alanine aminotransferase (ALT), aspartate aminotransferase (AST), albumin, total cholesterol, glucose and urea levels in colourimetric assays using a COBASs c111 analyser (Roche Diagnostics, Mannheim. Switzerland). Blood cell counts and haematological values were recorded with a DxH 800 (Beckman Coulter) automatic cell counter.

S3. Cell culture and treatment

3T3-L1 preadipocytes and C2C12 myoblast cells were grown in supplemented (10% foetal bovine serum (FBS), 1% penicillin/streptomycin) DMEM (DMEM-S) maintained in a 95% O_2,

5% CO_2 atmosphere at 37°C. The cells were seeded at a density of 40x10^4 cells/well in 24-well plates and allowed to reach confluence before treatment. The cells were exposed to 0.1, 0.25, 0.50, 0.80 or 1.0 mg/ml PSOn for 3, 6, 12, 24 or 48 h. Control cells were treated with PSOn solvent only. After treatment, the cells were evaluated for viability according to the quick protocol of the Vybrant MTT Cell Proliferation Assay (Thermo Fisher Scientific, USA). The absorbance of the control wells at 470 nm was considered to indicate 100% viability, and the viability of the treated cells was extrapolated accordingly.

S4. Primary astrocyte cell culture

Primary astrocytes were obtained from neonatal mice as previously described [1]. Briefly, postnatal P1-P4 pups were cleaned with ethanol and decapitated to obtain the brains. Under a stereoscopic microscope, the meninges were removed, and the cortices were separated and placed in cold Hank's balanced saline solution (HBSS). The cortices from 4 mice were collected and then triturated with scissors. The minced tissue was digested in 0.25% (final) trypsin solution for 30 min. The digested tissue was further mechanically homogenized using fire-polished Pasteur pipets. The cell suspensions were plated in 75 cm^2 culture flasks coated with poly-D-lysine (50 μg/ml). The cells were maintained in astrocyte culture medium (high-glucose DMEM (Gibco, Life Technologies, USA) supplemented with 10% heat-inactivated foetal bovine serum); after 7 days in culture, the microglia and oligodendrocyte precursors were detached by vigorous shaking. After intensive PBS washing, the cells were trypsinized and collected. The astrocyte-enriched cell suspensions were used to seed astrocytes at a density of 4x10^4 cells/well in astrocyte culture medium, and the cells were treated as described above.

3. Supplementary figure legends

Supplementary figure 1. Effect of acute treatment of PSOn at 0, 1, 2 and 4 mg/g body weight. A) Change in body weight of mice before and after a single PSOn administration. B) The ratio of

the indicated organ weight against body weight is plotted as a percentage for the control and PSOn-treated mice. C) Representative micrographs of liver (left panel) and kidney (right panel) histological H&E-stained tissue, showing the morphology at 20X and 40X (dotted lines) magnification. D) Quantification of blood levels of liver damage markers (ALT, AST, albumin) and lipid (triglycerides, cholesterol) and carbohydrate (glucose) metabolism indicators. *$p<0.05$.

Supplementary figure 2. Effect of chronic treatment of PSOn at 0, 8.7 mg/g, 26 mg/g and 35 mg/g. A) Change in body weight of mice before and after every third day of PSOn administration. B) The ratio of the indicated organ weight against body weight is plotted as a percentage for the control and PSOn-treated mice C) Representative micrographs of liver (left panel) and kidney (right panel) histological H&E-stained slides, showing the morphology at 20X and 40X (dotted lines) magnification. D) Quantification of blood levels of liver damage markers and lipid and carbohydrate metabolism. **$p<0.001$, ***$p<0.0001$ *vs*. control diet.

Supplementary figure 3. The viability of 3T3, C2C12 cells and primary astrocytes exposed to PSOn are not affected. A) Evaluation of cell viability using the MTT assay. The bars indicate the percentage of living cells at different doses. B) Representative micrographs of 3T3-L1 cells and primary astrocytes at 20X magnification. In both cases, the results of 3 independent experiments are shown.

Supplementary figure 4. Oxygen consumption rate (OCR) in mouse primary hepatocytes incubated for 3 h or 18 h (overnight) with PSOn in the presence or absence of oligomycin, carbonyl cyanide-p-trifluoromethoxyphenylhydrazone (FCCP) or rotenone. Data are the mean +/- SEM. No significant difference was found by one-way ANOVA.

4. Supplementary reference

1. Schildge S, Bohrer C, Beck K, Schachtrup C: Isolation and culture of mouse cortical astrocytes. *J Vis Exp* 2013(71).

Supplementary table 2. PCR primers sequences

Gene name	Gene ID	Forward	Reverse
**Aldehyde oxygenase 1 (*AOX-1*)**	NM_009676	CTCAGTGAACATCCCCTGGT	TGTGTCCAGCATCCCTTCAT
**Glutathione S-transferase, alpha 4 (*Gsta-4*)**	NM_010357	GAGGCTTTTCTCGTTGGCAA	CTTCCTCTGACTTCCGGGTT
**NADPH dehydrogenase, quinone 1 (*Nqo-1*)**	NM_008706	ACAGGTGAGCTGAAGGACTC	GTTGTCGTACATGGCAGCAT
**Nuclear factor, erythroid derived 2, like 2 (*Nfe-2l2*)**	NM_010902	CATGATGGACTTGGAGTTGC	CCTCCAAAGGATGTCAATCAA
**Glucose-6-phosphate-dehydrogenase X-linked (*G6pdx*)**	NM_008062	TGCCTTCCACCAAGCTGATA	GCTCACTCTGCTTTCGGATG
**Peroxiredoxin-1 (*Prdx-1*)**	NM_011034	GTGAGACCTGTGGCTCGAC	TGTCCATCTGGCATAACAGC
**Peroxisome proliferator activated receptor alpha (*Ppar-α*)**	NM_011144	CTGAGACCCTCGGGGAAC	AAACGTCAGTTCACAGGGAAG
**Peroxisome proliferator activated receptor beta (*Ppar-β*)**	NM_011145	CCATCCCAGCTTGTGGTC	GAGACAGCCTGGGAATATGG
**Peroxisome proliferator activated receptor gamma (*Ppar-γ*)**	NM_1127330	GAAAGACAACGGACAAATCACC	GGGGGTGATATGTTTGAACTTG
**Fatty acid synthase (*Fas-n*)**	NM_007988	CAACATGGGACACCCTGAG	GTTGTGGAAGTGCAGGTTAGG
**Sterol regulatory element-binding transcription factor 1 (*SREBP-1*)**	NM_011480	GCTTTCCCTTGAGGACCTTT	CTGATTGCTTGTCAGGCTCA
***18s* rRNA**	NR_003278	CGATTGGATGGTTTAGTGA	AGTTCGACCGTCTTCTCAG

Sequences are depicted in 5 prime to 3 prime directions.

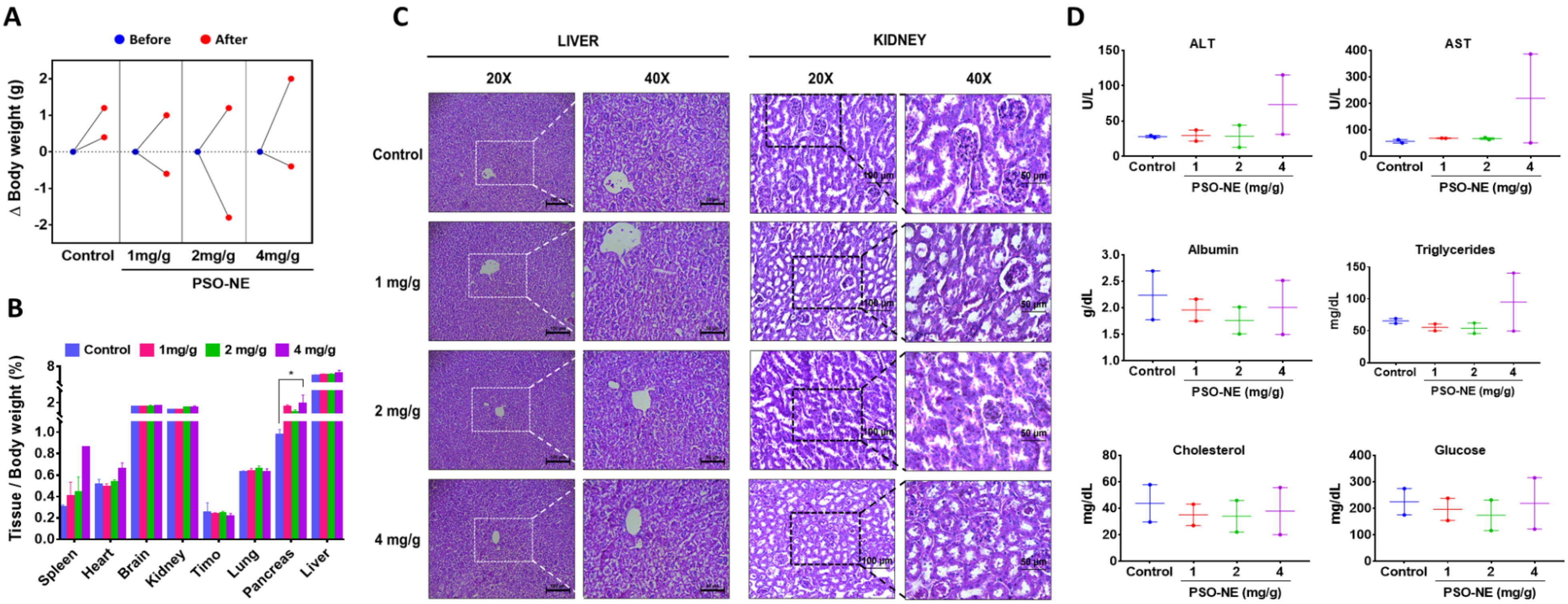

Supplementary figure 1

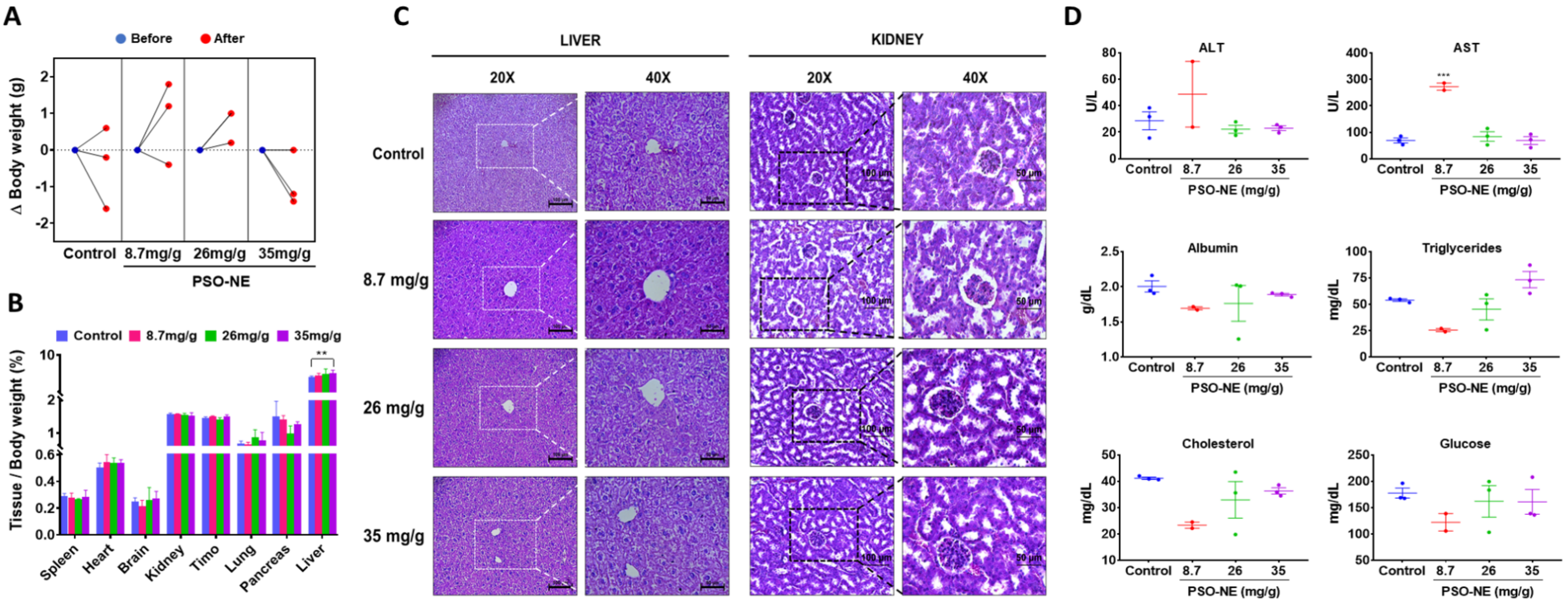

Supplementary figure 2

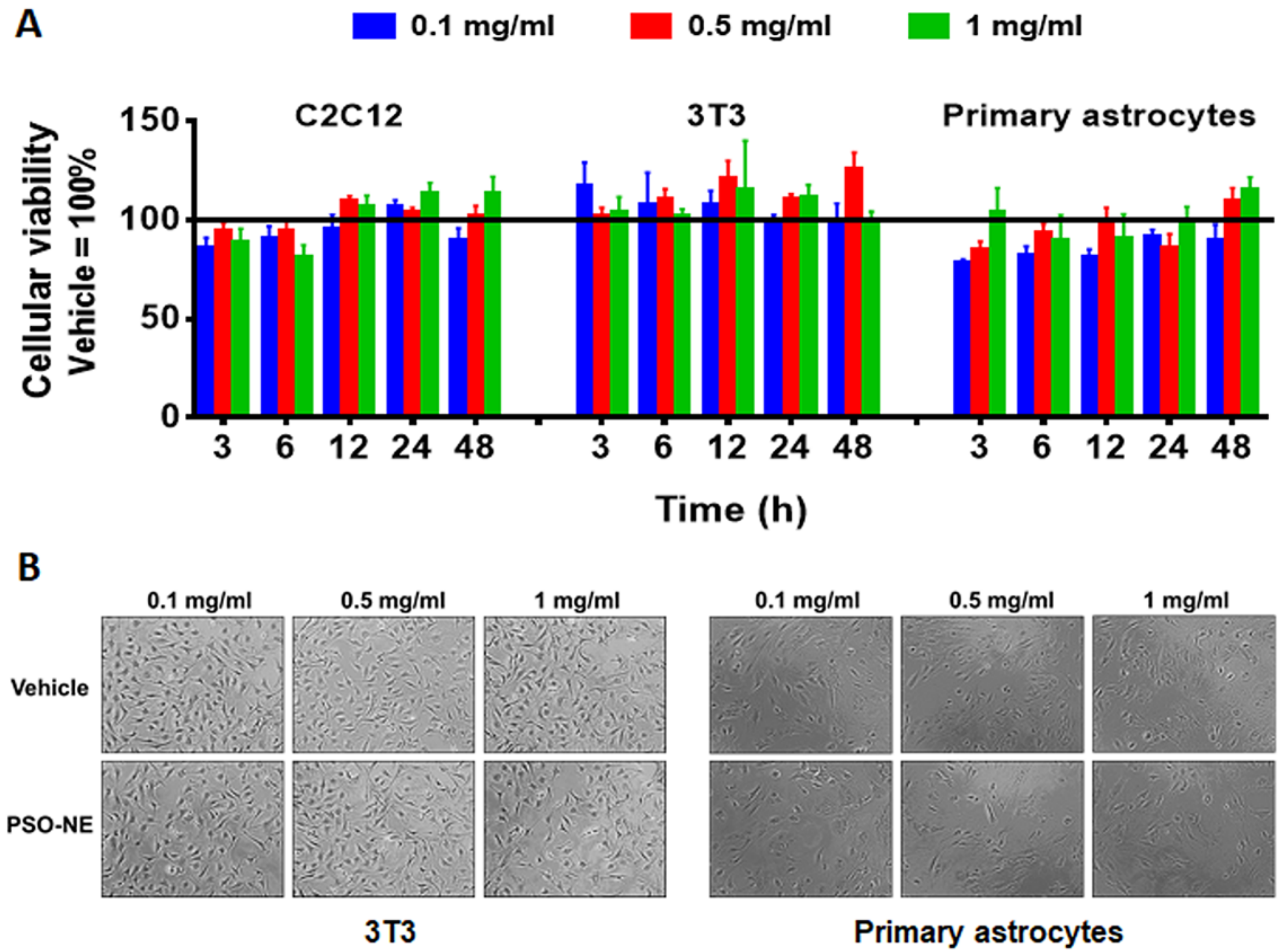

Supplementary figure 3

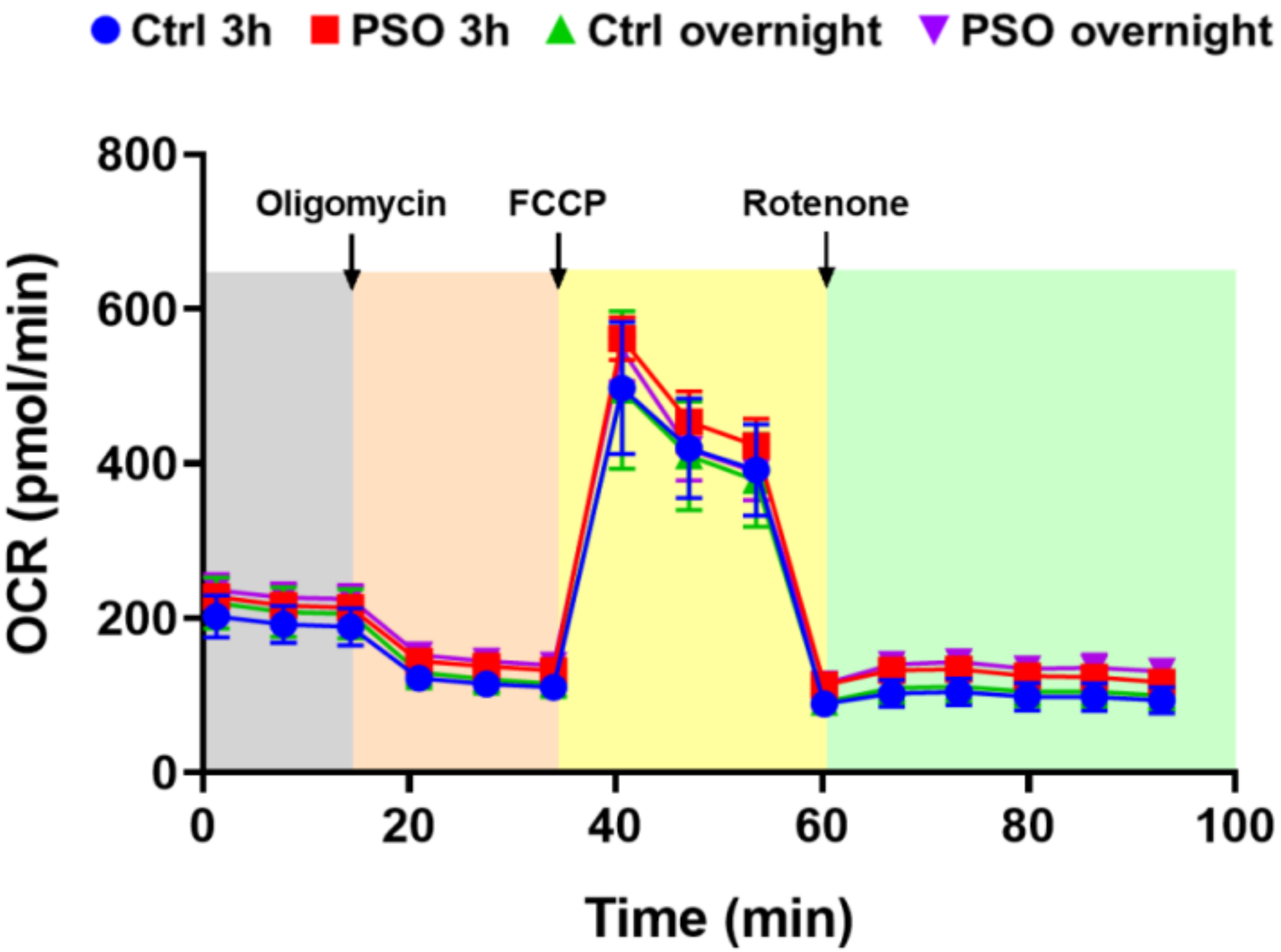

Supplementary figure 4
